# P45 Antimicrobial activity of ruthenium-based metallo-therapeutics against *Pseudomonas aeruginosa*

**DOI:** 10.1093/jacamr/dlac004.044

**Published:** 2022-02-16

**Authors:** Nicole S. Britten, Jonathan A. Butler

**Affiliations:** Manchester Metropolitan University, Manchester, UK; Manchester Metropolitan University, Manchester, UK

## Abstract

**Background:**

*Pseudomonas aeruginosa* is a Gram-negative opportunistic pathogen that is highly resistant to antibiotics and biocidal products used in both medical and industrial environments respectively. Metal-based compounds have been used as antimicrobial agents throughout history for a broad range of applications. More recently, it has been shown that ruthenium (Ru)-based compounds have potent antimicrobial properties and, in contrast to traditional antibiotics, these are thought to elicit antibacterial activity at multiple sites within the bacterial cell, which will undoubtedly reduce the possibility of resistance evolution.

**Methods:**

MIC and MBC assays coupled with disc diffusion assays were used to screen a library of Ru-based compounds.

**Results:**

One lead compound was identified that was highly active at inhibiting growth of multiple strains of *P. aeruginosa* at ≤32 mg/L. Crystal violet biofilm assays were performed, which showed a decrease in biomass following exposure over a 24 h period. Scanning electron microscopy was used to reveal morphological changes in the bacterial cell ultrastructure when exposed to the Ru-based compound, with evidence of membrane perturbation that supported a proposed mechanism of antimicrobial activity.

**Conclusions:**

These findings make a significant contribution towards the search for novel bactericidal agents and further research is now focused on determining the potential for use as novel adjuvants within medicinal applications such as wound care management.

